# Microstructure, optical properties, and catalytic performance of Cu_2_O-modified ZnO nanorods prepared by electrodeposition

**DOI:** 10.1186/s11671-015-0755-0

**Published:** 2015-01-31

**Authors:** Xishun Jiang, Qibin Lin, Miao Zhang, Gang He, Zhaoqi Sun

**Affiliations:** School of Physics and Material Science, Anhui University, Hefei, 230601 China; School of Electronic and Electrical Engineering, Chuzhou University, Chuzhou, 239000 China

**Keywords:** ZnO nanorods, Microstructure, Optical properties, Catalytic performance

## Abstract

Cu_2_O-modified ZnO nanorods are prepared by a two-step electrodeposition method on ITO substrates, and the deposition time of Cu_2_O is 0, 1, 5, and 10 min, respectively. Cu_2_O particles are embedded in the interspaces of the ZnO nanorods, and the amounts of the Cu_2_O particles increase obviously when the deposition time lasts longer. The peaks corresponding to ZnO nanorods and Cu_2_O particles are detected from scanning electron microscope (SEM) and X-ray diffraction (XRD) results. UV-vis absorption spectra measurements have shown the bandgaps of ZnO nanorods shift from 3.22 to 2.75 eV. The methyl orange (MO) concentration can be reduced to around 15% in 100 min with Cu_2_O electrodeposition time for 10 min.

## Background

Zinc oxide (ZnO), a typical n-type semiconductor with a direct bandgap of 3.37 eV, is an attractive material that could be suitable for a window layer [1-4]. Low-dimensional nanostructural ZnO shows unique physical and chemical properties [5-8]. Up to now, great attention has been focused on the investigation of ZnO nanostructures including thin film, nanowires, nanorods, and nanoparticles [9-13]. Due to the high specific surface area and excellent optical and electrical properties, ZnO nanorods have attracted much attention for their applications in solar cells [14-18]. Unfortunately, the absorption of ZnO in the visible-light region is very low due to its wide bandgap [19,20]. To extend the absorption of ZnO into the visible region, narrow bandgap semiconductors, such as CdS, CdSe, and Cu_2_O, have been used to construct heterostructures with 1D ZnO [21-24]. Cuprous oxide (Cu_2_O), with a direct bandgap of 2.17 eV, is a natural p-type semiconductor owning good mobility and high minority carrier diffusion length [25,26]. Low-cost producibility, abundance, nontoxicity, and high absorption coefficient in the visible light region make Cu_2_O a promising material for photovoltaic application [27,28]. Cu_2_O is considered to be a promising partner with ZnO for p-n heterojunction due to its narrow energy band [29,30].

The Cu_2_O/ZnO heterojunction has always been synthesized by several methods, such as thermal oxidation, sputtering, pulsed laser deposition, chemical vapor deposition, and electrodeposition. To our knowledge, much attention is focused on the Cu_2_O/ZnO heterojunction for solar cells, and there are few reports involving Cu_2_O/ZnO nanorods for photocatalysis studies [31-33]. Jeong et al. [34] reported that interface recombination is the dominant carrier transport mechanism, and Cu_2_O/ZnO heterojunction solar cells have high potential as solar cells if the recombination and tunneling at the interface can be suppressed at room temperature. In the current work, we prepared Cu_2_O-modified ZnO nanorods by a two-step electrodeposition method. The amount of Cu_2_O is controlled by the deposition time. The effects of the deposition time on the morphological, microstructural, optical properties, and catalytic performance of the Cu_2_O-modified ZnO nanorods have been investigated in detail.

## Methods

### Preparation of Cu_2_O-modified ZnO nanorods

The Cu_2_O-modified ZnO nanorods were prepared by a two-step electrodeposition method on ITO substrates. Prior to the deposition, ITO substrates were ultrasonically cleaned in acetone, alcohol, and deionized water, sequentially. Firstly, an aqueous solution composed of 0.01 mol/L zinc nitrate (Zn(NO_3_)_2_) was used to prepare ZnO nanorods. The ITO substrates served as the working electrode, platinum worked as the counter electrode, and an Ag/AgCl electrode was the reference electrode. The electrodeposition procedure was conducted in a water bath for 1 h at a temperature of 70°C under the constant potential of −0.7 V vs the reference electrode. Consequently, the electrochemical deposition of Cu_2_O on the ZnO nanorods was performed in an aqueous solution composed of 0.05 mol/L copper acetate (Cu(CH_3_COO)_2_) and 0.1 mol/L sodium acetate (NaCH_3_COO). The ZnO/ITO films served as the working electrode, and the temperature of water bath was 40°C. The applied potential was controlled at −0.2 V vs the reference electrode and lasted for different times (1, 5, and 10 min) for each Cu_2_O-modified ZnO nanorods. The samples were labeled as Cu_2_O(1)-ZnO, Cu_2_O(5)-ZnO, and Cu_2_O(10)-ZnO, respectively. The pure Cu_2_O film was deposited at the same condition, and the deposition time is 30 min.

### Characterization

The phase and crystalline structure of the as-deposited films were examined by X-ray diffraction (XRD; MAC Science, Yokohama, Japan) with an X-ray diffractometer employing Cu-Kα radiation. The surface morphology of the Cu_2_O-modified ZnO nanorod films was observed with a field-emission scanning electron microscope (FESEM; S4800, Hitachi, Ltd., Chiyoda, Tokyo, Japan). A UV-visible (UV-vis) spectrophotometer (UV-2550, Shimadzu, Tokyo, Japan) was used to measure the UV-vis absorption spectra of the as-deposited films. The surface composition was analyzed by X-ray photoelectron spectrometer (XPS; ESCALAB 250, Thermo Fisher Scientific, Waltham, MA, USA). The Raman spectra and photoluminescence (PL) spectra were recorded by micro-Raman spectroscope system. The photocatalytic activity of the as-prepared samples was evaluated by the photodegradation of methyl orange (MO) solution under visible light irradiation. The visible light source was obtained using a 420-nm cutoff filter. The samples (15 mm × 10 mm) were immersed in 10 mL 15 ppm MO solutions. The distance between the samples and the light source was fixed at 5 cm. After the given time interval, the UV-vis absorption spectra of MO were recorded by UV-vis spectrophotometer (Shimadzu, UV-2550).

## Results and discussion

### Surface morphology analysis

**Figure 1:**
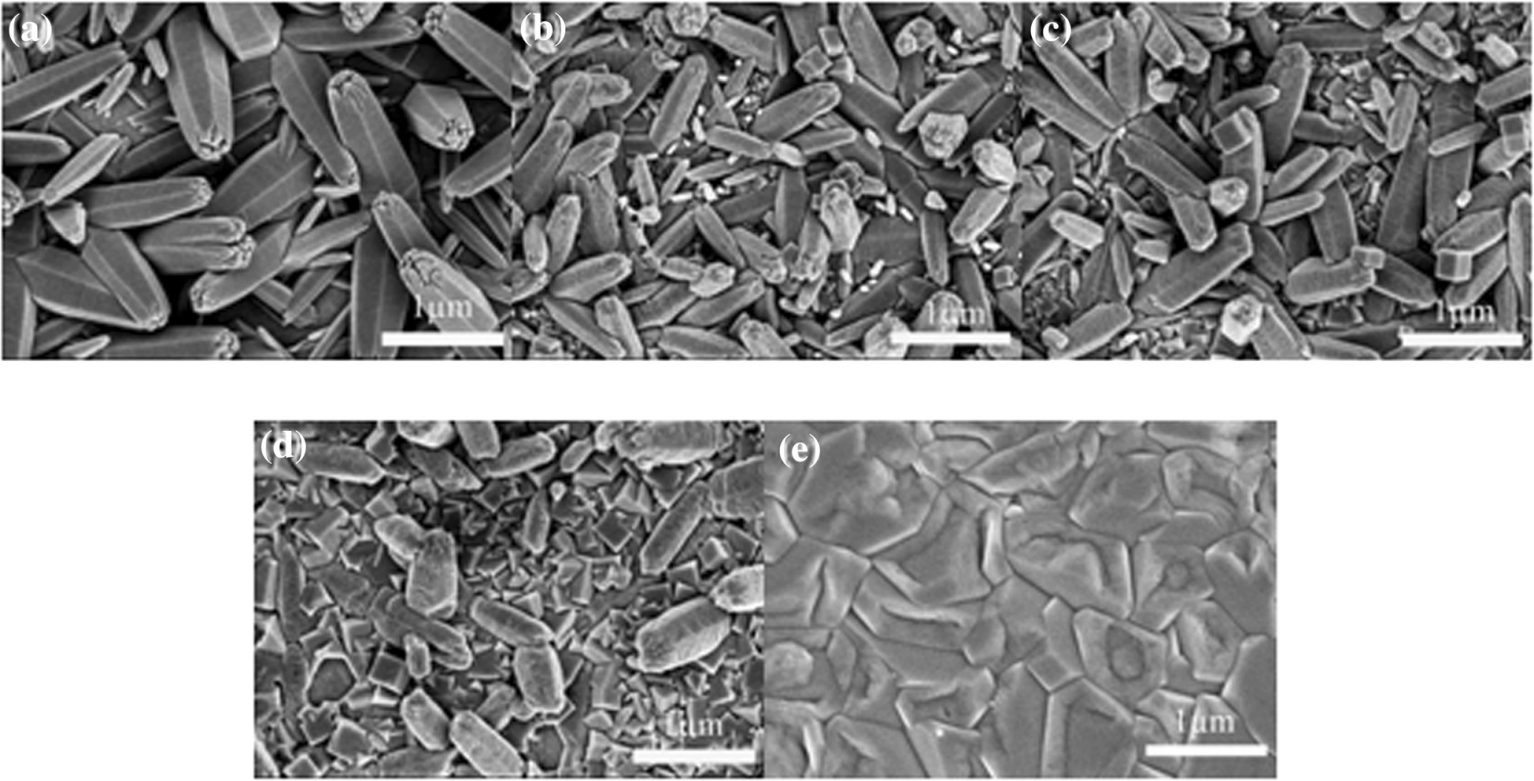
**SEM micrographs of the Cu**_**2**_ **O-modified ZnO nanorods.** Cu_2_O-ZnO modified with different Cu_2_O deposition times of **(a)** 0 min, **(b)** 1 min, **(c)** 5 min, and **(d)** 10 min and **(e)** pure Cu_2_O, respectively.

Figure 1 shows the scanning electron microscope (SEM) micrographs of the Cu_2_O-modified ZnO nanorods with different Cu_2_O deposition times. As shown in Figure 1a, each hexagonal nanorod has a diameter of about 200 nm and the length is about 1 μm. The nanorods are gradient and uniformly disperse on the ITO substrates. From Figure 1, it can be observed that cubic structure Cu_2_O particles embedded in the interspaces of the ZnO nanorods and the amounts of the Cu_2_O particles increase obviously when the deposition time increases [35]. It can also be found that as the Cu_2_O deposition time increases, the diameter and length of the nanorods decreased, which can be affected by electrolyte corrosion during the Cu_2_O deposition process.

### Microstructure analysis

**Figure 2:**
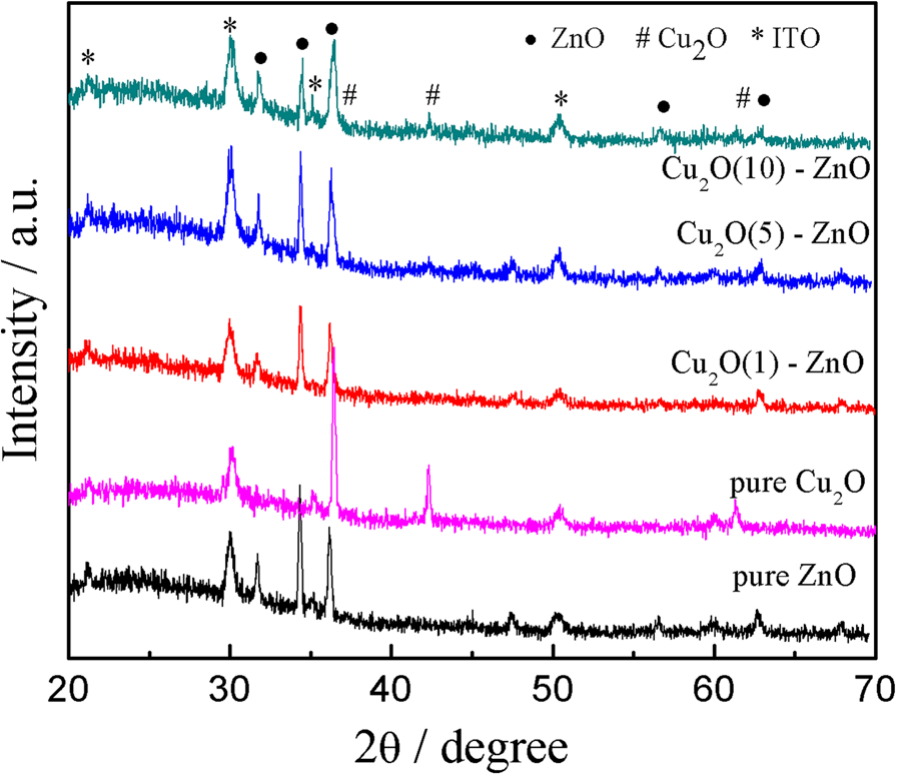
**XRD patterns of the Cu**_**2**_ **O-modified ZnO nanorods.**

Figure 2 illustrates the XRD pattern of the Cu_2_O-modified ZnO nanorods with different deposition times of Cu_2_O particles. From Figure 2, the characteristic peaks of Cu and CuO are not observed for all the samples, suggesting that no metallic copper or CuO formed in the electrodeposition process. The single-phase polycrystalline Cu_2_O films have been obtained only with the applied potential below −0.3 V [36]. In Figure 2a, apart from the diffraction peaks corresponding to the ITO substrate, the peaks that corresponded to the reflections are 100, 002, 101, 102, 110, and 103 peaks of ZnO nanorods according to JCPDS: 89-1397. In Figure 2b,c,d, besides the peaks of ZnO nanorods and ITO substrate, the diffraction peaks of 111, 200, and 220 crystal planes of Cu_2_O appear (JCPDS: 05-0667). The Cu_2_O (111) peak (2*θ* = 36.50°) is very close to the ZnO (101) peak (2*θ* = 36.25°), and they are overlapped in the pattern. The intensities of the Cu_2_O characteristic peaks increase with the Cu_2_O electrodeposition time for increased amounts of the Cu_2_O nanoparticles. The characteristic peaks of Cu_2_O electrodeposited for 1 min (Figure 2b) can barely be detected, and this can be ascribed to an insufficient amount. In a word, the peaks of Cu_2_O particles are relatively weaker due to the shorter deposition time compared with ZnO nanorods.

### UV-vis absorbance and XPS analysis

**Figure 3:**
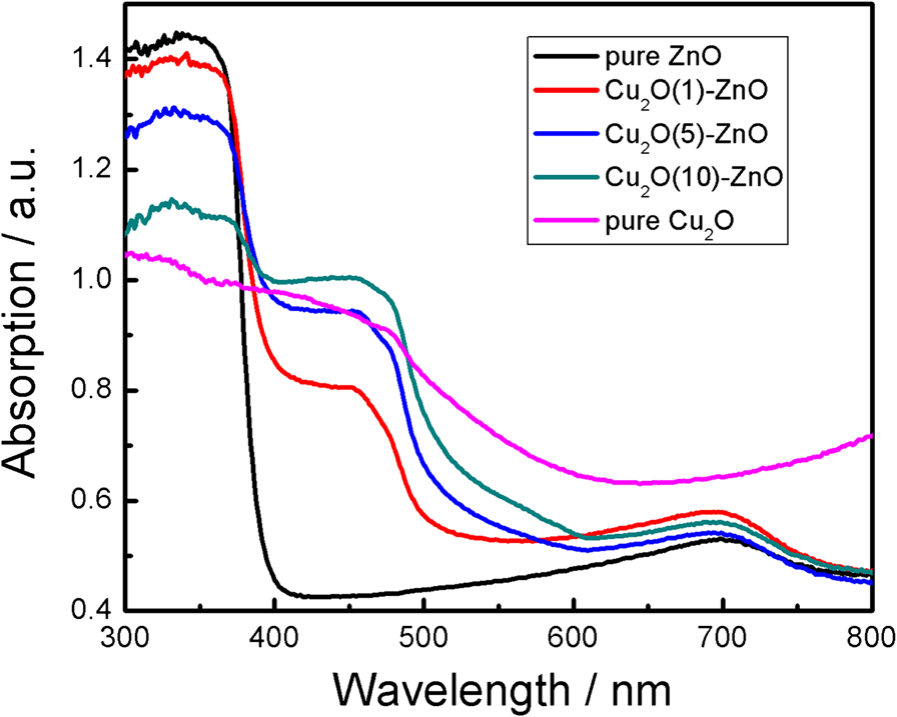
**UV-vis absorption spectra of the samples.**

Figure 3 shows the optical absorption spectra for the Cu_2_O-modified ZnO nanorods with different Cu_2_O deposition times from 0 to 10 min. An absorption edge at 390 nm for the ZnO nanorods was observed, as shown in Figure 3a. The absorption edges of the Cu_2_O-modified ZnO nanorods show an obvious redshift compared with pure ZnO nanorods and exhibit a broad absorption band in the UV region, which originates from the combinational effect of the narrow bandgap of Cu_2_O (approximately 2.17 eV) and wide bandgap of ZnO (approximately 3.37 eV) [37]. The absorbance in the visible light range increases with the increase of the deposition time of Cu_2_O. The introduction of Cu_2_O particles in ZnO nanorods extends the absorption edge to the visible light range, which is very important in making full use of sunlight.

**Figure 4:**
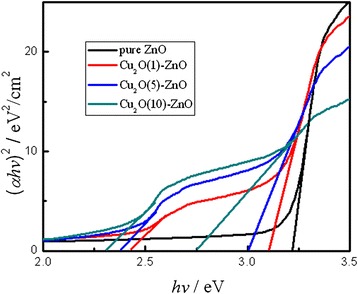
**Plot of (** ***ahv*** **)** ^**2**^ **vs photon energy for Cu**_**2**_ **O-modified ZnO nanorods.**

The optical bandgaps of ZnO and Cu_2_O can be determined based on the equation: (*αhν*)^2^ = *A*(*hν* − *E*_g_) [38]. The energy bandgap (*E*_g_) is measured by linear extrapolation to the *hv*-axis. The inset of Figure 4 shows (*ahv*)^2^ versus *hv* for the Cu_2_O films, and the estimated direct bandgaps are listed in Table 1. Due to variation in deposition time, the value of absorption spectra changed. For Cu_2_O-modified ZnO nanorods, when the Cu_2_O deposition time increases from 1 to 10 min, the corresponding bandgaps of Cu_2_O particles are 2.43, 2.38, and 2.30 eV, respectively. In addition, the bandgaps of ZnO nanorods shift from 3.22 to 2.75 eV, which is also consistent with previous SEM and XRD results.

**Table 1:** **The estimated direct bandgaps of the Cu**_**2**_ **O-modified ZnO nanorods**

	**Cu**_**2**_ **O deposition time (min)**			
	**0**	**1**	**5**	**10**
Cu_2_O bandgap (eV)	-	2.43	2.38	2.30
ZnO bandgap (eV)	3.22	3.11	3.00	2.75

**Figure 5:**
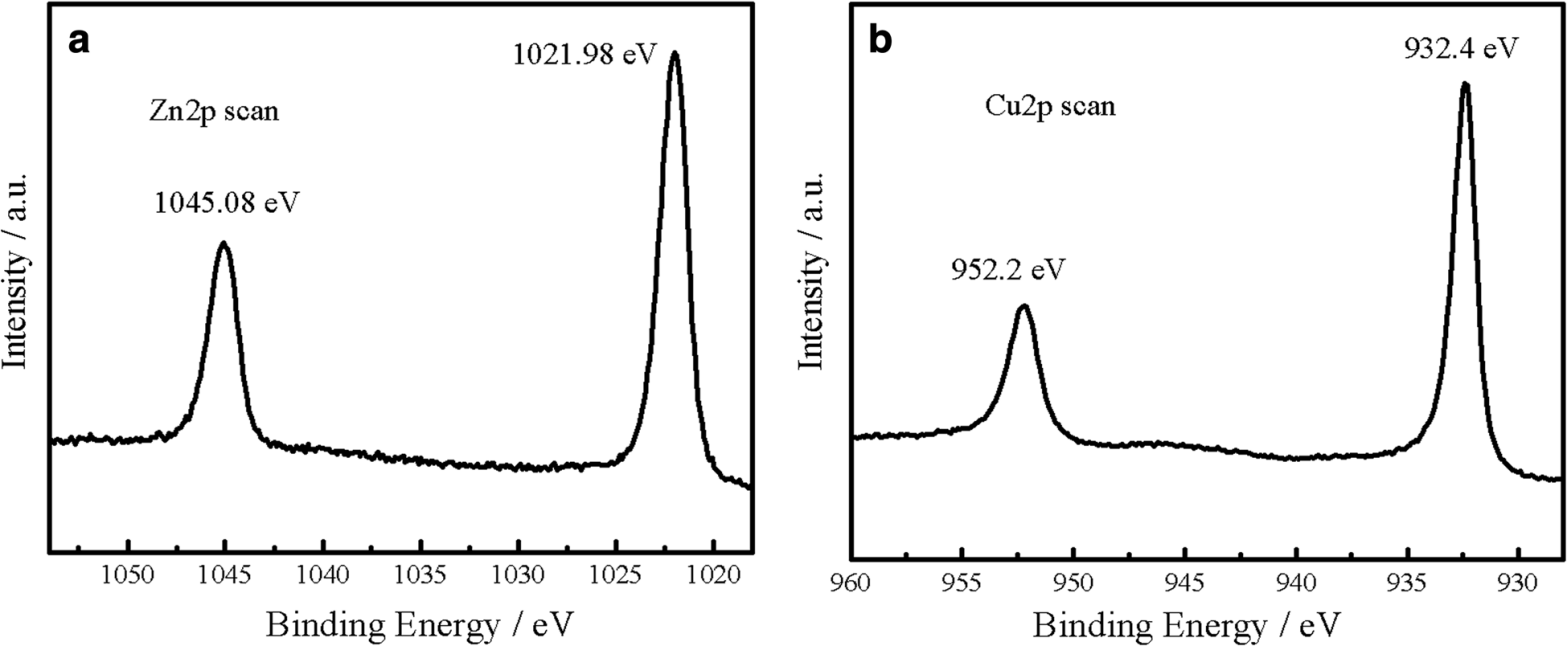
**XPS spectra of the sample with Cu**_**2**_ **O deposition for 5 min (a, b).**

XPS measurements were conducted for surface analysis of the Cu_2_O-modified ZnO nanorods with Cu_2_O deposition time for 5 min (Figure 5). As shown in Figure 5, the peaks of Zn2*p* and Cu2*p* are detected from the XPS spectrum. Two peaks of Zn2*p* located at 1,045.1 and 1,021.9 eV are assigned to Zn2*p*3/2 and Zn2*p*1/2, respectively, which can be assigned to Zn^2+^ in ZnO nanorods (Figure 5a) [39]. The typical XPS peaks of Cu (2*p*) at 952.2 and 932.4 eV for the Cu_2_O-modified ZnO nanorods indicate the existence of Cu^+^ during the deposition of Cu_2_O particles (Figure 5b). Furthermore, the characteristic peaks for Cu^2+^ at 953.6 (2*p*1/2) and 933.7 eV (2*p*3/2) were not observed [40]. This result confirms that the sample contains Cu^+^ rather than Cu^2+^ or Cu.

### Raman and PL spectra analysis

**Figure 6:**
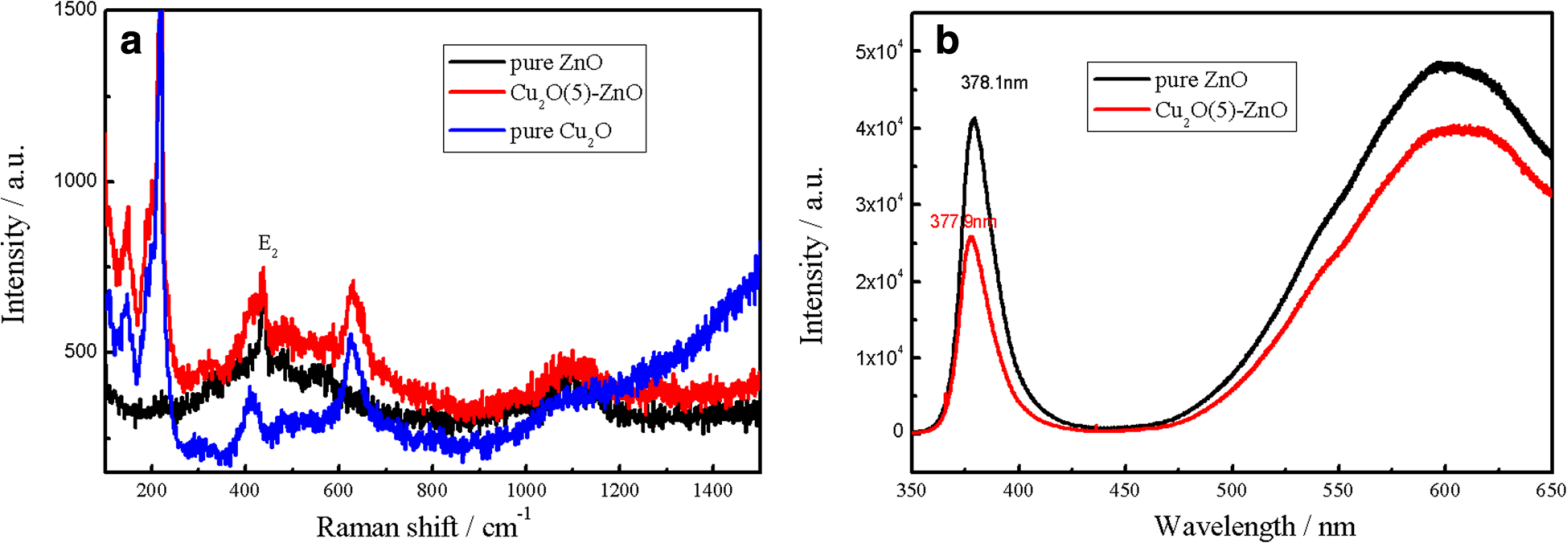
**Raman (a) and PL (b) spectra of the sample with Cu**_**2**_ **O deposition for 5 min.**

Figure 6 shows the Raman and PL spectra of Cu_2_O-modified ZnO nanorods with Cu_2_O deposition for 0 and 5 min. The peak at 437 cm^−1^ is due to the *E*_2_ high vibration mode of ZnO hexagonal crystal structure as shown in Figure 6a [41]. Figure 6a also shows that the stronger Raman peak of 218 cm^−1^, which corresponds to 2Γ_12−_ vibration modes of Cu_2_O. Meanwhile, the relative weaker peaks of 146 and 626 cm^−1^ can be contributed to infrared vibration mode Γ_15_, which are excited from oxygen vacancy [42]. Figure 6b shows the PL spectra of Cu_2_O-modified ZnO nanorods. There appeared two component peaks of the UV emission in the PL spectra of Cu_2_O-modified ZnO nanorods. The predominant sharp peak appeared at about 380 nm could be assigned to the near band emission of the ZnO nanorods [43]. In addition, a wide emission with a peak at 600 nm was detected and regarded as defect-related emissions of ZnO nanorods. The existence of Cu_2_O particles has little impact on peak position of ZnO nanorods. The emissions at 380 and 600 nm were diminished when Cu_2_O particles were deposited on the ZnO nanorods, which may originate from the random multiple scattering in such structure.

### Photocatalytic degradation of MO

**Figure 7:**
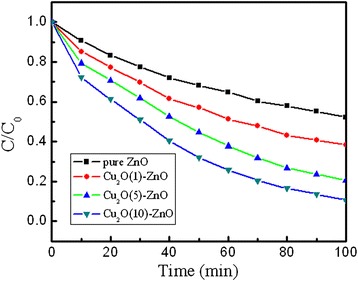
**The visible light photocatalytic degradation ratios to MO of the samples.**

The photocatalytic activities of the as-prepared samples were carried out by the degradation of MO solution under visible light irradiation, and the experimental results are shown in Figure 7. Here, C_0_ and C are the absorbance of the characteristic absorption peak (464 nm) of MO solution before and after irradiation. As indicated in Figure 7, the pure ZnO nanorods exhibit a weak ability for the degradation of MO. The poor degradation ability of the pure ZnO nanorods can be ascribed to the fact that the visible light cannot provide energy to excite electrons from the valance band to the conduction band. All the Cu_2_O-modified ZnO nanorods have strong degradation ability of MO than the pure ZnO nanorods [44]. With increasing Cu_2_O electrodeposition time, the degradation abilities of the Cu_2_O-modified ZnO nanorods enhanced. The reason is that Cu_2_O has higher degradation ability than ZnO. Meanwhile, the amount of Cu_2_O particles on the ZnO nanorods increases when increasing the Cu_2_O electrodeposition time. Furthermore, the Cu_2_O-modified ZnO nanorods have a large specific surface area than pure ZnO nanorods. It is worth mentioning that the MO concentration can be reduced to around 15% in 100 min with Cu_2_O electrodeposition time of 10 min. As a result, the photocatalytic activity of the Cu_2_O-modified ZnO nanorods depends on the Cu_2_O electrodeposition time.

## Conclusions

In summary, the Cu_2_O-modified ZnO nanorods are prepared by electrodeposition method on ITO substrates. XRD measurement shows the coexistence of Cu_2_O with cubic structure and ZnO with hexagonal structure. SEM images reveal that Cu_2_O particles embed in the interspaces of the ZnO nanorods and the amounts of the Cu_2_O particles increase obviously when the Cu_2_O deposition time lasts longer. The absorbance in visible light range increases with the increase of the deposition time of Cu_2_O. All the Cu_2_O-modified ZnO nanorods have strong degradation ability of MO than the pure ZnO nanorods under visible light irradiation. The nanorod's structure of the ZnO had been broken and resulted to a significant decrease of the special surface area associating with the increase of the Cu_2_O deposition time. The obtained films may be used in fabricating solar cell and treating dye wastewater.
